# Involvement of the Opioid Pathway in the Antinociceptive Activity of the Aqueous Stem Extract from *Doyerea emetocathartica*

**DOI:** 10.3390/pharmaceutics18080952

**Published:** 2026-07-31

**Authors:** Genil Dantas de Oliveira, Magna Maria Lima Araújo, Paulo César Dantas da Silva, Gabriela Ribeiro de Sousa, Mariana França de Moraes, Wêndeo Kennedy Costa, Maria Tereza dos Santos Correia, Alisson Macário de Oliveira, Samuel Paulo Cibulski, Harley da Silva Alves

**Affiliations:** 1 Programa de Pós-Graduação em Ciências Farmacêuticas, Departamento de Farmácia, Universidade Estadual da Paraíba, Campina Grande 58429-500, PB, Brazil; genil.dantas.oliveira@aluno.uepb.edu.br (G.D.d.O.); magna.araujo@aluno.uepb.edu.br (M.M.L.A.); paulodantas@servidor.uepb.edu.br (P.C.D.d.S.); g.ribeiro@visitante.uepb.edu.br (G.R.d.S.); samuel.cibulski@ufrn.br (S.P.C.); 2 Graduação em Farmácia, Departamento de Farmácia, Universidade Estadual da Paraíba, Campina Grande 58429-500, PB, Brazil; mariana.franca@aluno.uepb.edu.br; 3 Departamento de Bioquímica, Universidade Federal de Pernambuco, Recife 50670-901, PE, Brazil; wendeocosta@gmail.com (W.K.C.); maria.tscorreia@ufpe.br (M.T.d.S.C.); 4 Departamento de Farmácia, Universidade Federal do Rio Grande do Norte, Natal 59012-570, RN, Brazil; alisson.macario@ufpe.br; 5 NuBioCaatinga, Núcleo de Bioprospecção da Caatinga, Universidade Federal do Rio Grande do Norte (UFRN), Santa Cruz 59200-000, RN, Brazil

**Keywords:** ethnopharmacology, cabeça de negro, HPLC analysis, cayaponoside D2, pain, analgesic activity, opioids receptors

## Abstract

**Background:** *Doyerea emetocathartica* (Cucurbitaceae) is endemic to the Brazilian Caatinga, commonly known as “cabeça-de-negro”, and is traditionally used to treat various conditions, including pain. Previous studies have demonstrated that the aqueous stem extract exhibits anti-inflammatory activity, which may be associated with the presence of cayaponoside D2, a major component of this extract. Given this, the aim of this study was to characterize the aqueous extract of *D. emetocatharthica* stems in terms of its Cayaponoside D2 content and to evaluate its antinociceptive activity using in vivo models, as well as to assess the involvement of the opioid pathway in the observed effect. **Methods:** For this purpose, the extract was analyzed by HPLC to quantify cayaponoside D2, and formalin- and acetic acid-induced nociception models were used to evaluate antinociceptive activity; the rats were divided into six groups (n = 6). The test groups received the aqueous extract of the *D. emetocathartica* stem (DEAQ) at doses of 25, 50, and 100 mg/kg; the control group received 0.9% saline; and morphine and indomethacin were used as positive controls. To investigate the mechanism of action involved in the antinociceptive effect, an evaluation was conducted of the role of the opioid pathway and the involvement of opioid receptors in the antinociceptive effect in the presence of specific antagonists: naloxone, CTOP, naltrindole, and Nor-BNI. **Results:** The analgesic activity of DEAQ was demonstrated in acetic acid-induced abdominal writhing and formalin-induced nociception models, with the most pronounced effects observed at doses of 50 and 100 mg kg^−1^. In the formalin test, DEAQ showed a predominant effect during the inflammatory phase. Mechanistic investigations revealed that the antinociceptive activity involves the opioid system, mainly through μ-opioid receptors, with additional participation of δ-opioid receptors, as evidenced by the partial reversal of the effect by selective antagonists. These findings suggest that DEAQ may exert a multimodal analgesic effect involving both opioid and non-opioid pathways. This activity can be attributed to the presence of cayaponoside D2, which was quantified in the extract at a concentration of 108 mg g^−1^ of dry extract. **Conclusions:** These findings demonstrate that the aqueous stem extract from *D. emetocathartica* exhibits promising analgesic activity mediated by the opioid pathway, reinforcing its pharmacological potential and supporting its traditional use in pain management.

## 1. Introduction

Pain is defined as an unpleasant sensory and emotional experience and may be associated with actual or potential tissue damage. This condition can be caused by various stimuli—whether biological, mechanical, social, or psychological—and its perception can vary from person to person, characterizing it as an individual and multidimensional experience [1]. Physiologically, pain is one of the inflammatory signals resulting from the inflammatory process, alerting the individual to the presence of harmful stimuli, minimizing the risk of tissue damage, and protecting against further tissue damage [2].

The duration of the pain allows for classification into acute pain, which occurs rapidly and intensely, signaling damage or a threat to the body—it can be caused by various conditions (illness, surgery, trauma), typically lasting less than six months and ceasing once the underlying cause is treated—and chronic pain, resulting from a current or previously treated health condition, with the painful sensation persisting or recurring for longer than a response to an initial stimulus for a period exceeding three months [3]. Chronic pain affects approximately 30% of the world’s population and constitutes a major factor in comorbidity, with a significant impact on physical, mental, and social health [4].

In addition, the type of stimulus sent or detected by sensory neurons classifies pain as nociceptive, neuropathic, or neuroplastic. Neuropathic pain directly involves injury or disease of somatosensory neural tissue, as it occurs in certain conditions such as systemic sclerosis and paraneoplastic neuropathy. Neuroplastic or nociplastic pain results from a sensory dysfunction that amplifies the sensation of pain, showing no clear mechanistic relationship between tissue injury (neural or non-neural) and pain intensity; it can be localized or diffuse, sometimes triggered by non-painful stimuli such as sounds, odors, and lights, as occurs, for example, in fibromyalgia [5]. Nociception, in turn, is defined as the neurophysiological process that detects and encodes a specific harmful stimulus to non-neural tissue, which causes tissue damage or has the potential to do so. This stimulus may be thermal, mechanical, chemical, or electrical and is detected by the peripheral nervous system via nociceptors—receptors sensitive to these painful stimuli—with subsequent transduction of the signal to the spinal cord and interpretation of the signal by the brain, which generates the sensation of pain [6].

The therapeutic management of pain is broad and diverse, including drugs that act centrally and peripherally, such as opioid analgesics, anticonvulsants, and serotonin and norepinephrine reuptake inhibitors, as well as classes such as steroidal and nonsteroidal anti-inflammatory drugs. However, inappropriate or prolonged use of these medications can lead to dependence or damage to the cardiovascular, respiratory, nervous, and digestive systems [7,8]. Given this, natural products exhibit analgesic potential and serve as sources for the main drugs used in current clinical practice, such as morphine and codeine. In view of the need for analgesic agents with novel, more effective, and safer mechanisms of action, such agents are both necessary and promising. To this end, phytochemical studies combined with clinical and preclinical testing are essential for evaluating pharmacological properties [9].

Plants of the Cucurbitaceae family are characterized by the production of highly oxygenated tetracyclic triterpenes known as cucurbitacins; these substances exhibit diverse pharmacological potential, including hepatoprotective activity, liver protection, anticancer, anti-inflammatory, antiviral, and anti-diabetic properties [10]. Furthermore, several studies have demonstrated the modulatory potential of some species within this family on nociception and inflammatory pain, including *Cucumis sativus*, *Cucumis ficifolius*, and *Trichosanthes kirilowii* [11,12,13,14]. The presence of cucurbitane-type triterpenoid saponins is particularly abundant and noteworthy in species of this family, exhibiting a diverse structural range with significant bioactivities, including antimicrobial, anti-inflammatory, anti-diabetic, and anti-tumor properties [15].

Many species within this family have been associated with traditional use for the treatment of inflammation and pain, with emphasis on *Doyerea emetocathartica* Grosourdy grows in the Brazilian Caatinga, and are traditionally used to treat skin conditions, rheumatism, and for pain relief. Previous studies conducted by the research group demonstrated that the aqueous extract of the stems exhibited no acute toxicity or genotoxicity and showed anti-inflammatory potential through the modulation of cytokines and immune system cells, which may be related to the chemical profile of the aqueous extract from *D. emetocathartica*, which revealed that the triterpene saponin, named cayaponoside D2, is the main component of this extract [16]. Although several studies have demonstrated the antinociceptive potential of these compounds through the modulation of inflammatory responses, investigations evaluating the mechanisms involved in pain control through other pathways, such as the opioid system, remain scarce [17].

Given these findings, further investigation of the pharmacological potential of the stem extract from *D. emetocathartica* in nociception models is warranted, as modulation of the inflammatory response may contribute to the reduction of nociceptive sensitization. Furthermore, elucidating the underlying mechanisms of action is essential to better understand the therapeutic potential of this species and its possible application in pain management.

## 2. Materials and Methods

### 2.1. Chemicals

All solvents used for HPLC analysis, including methanol (MeOH) and formic acid, were of HPLC grade and obtained from commercial suppliers. Phosphoric acid (H_3_PO_4_) and ultrapure water were used for mobile phase preparation and sample dilution. Acetic acid and formalin were purchased from standard commercial sources and were of analytical grade. Morphine, indomethacin, naloxone, CTOP, naltrindole, and nor-BNI were obtained from Sigma-Aldrich (St. Louis, MO, USA). The cayaponoside D2 standard used for HPLC quantification was a secondary standard, previously isolated and structurally characterized by our research group in earlier studies [16].

### 2.2. Quantification of Cayaponoside D2 of DEAQ by HPLC Analysis

This work is a continuation of previous investigations conducted by our research group. The procedures related to plant material collection, sample preparation, and ex-traction have been described in detail in Oliveira et al. (2025) [15]. The extract previously obtained was used without modification in the present study. Hereafter, the aqueous extract obtained of the stems from *D. emetocathartica* is referred to as *D. emetocathartica aqueous* (DEAQ), and this abbreviation is used consistently throughout the manuscript.

The method used for the quantification of cayaponoside D2 in DEAQ was performed by HPLC using a Shimadzu^®^ high-performance liquid chromatograph equipped with two LC-20AD pumps, an SIL-20A-HT autosampler, a CTO-20A column oven, an SPD-20A UV/Vis detector, a CBM-20A system controller, and a computerized data processing system with LC Solution^®^ software. Chromatographic analyses were carried out using a reverse-phase C18 column maintained at 40 °C. The mobile phase consisted of water containing 0.1% formic acid (A) and methanol (B), using a gradient elution from 50 to 100% B over 15 min, followed by re-equilibration prior to the next analysis. The flow rate was set at 1.0 mL min^−1^, and the injection volume was 10 µL. Detection was performed at 239 nm, selected based on the maximum absorbance of cayaponoside D2. In addition, extract and standard samples were made in triplicate and filtered with the diluent solution at a concentration of 50%: 50% MeOH:acidified water (0.1% of H_3_PO_4_).

For quantitative analysis, a calibration curve was constructed using a previously characterized cayaponoside D2 standard in the concentration range of [12–200 µg mL^−1^] using linear least-squares regression, and the correlation coefficient (R^2^) was used to confirm linearity. Results were expressed as mg of cayaponoside D2 equivalents per g of dry extract.

### 2.3. Animals

Adult male Swiss mice aged around 60 days and weighing between 30 and 35 g were obtained from the Keizo Asami Institute (Recife, Pernambuco, Brazil). The animals were acclimatized for 120 h in a controlled environment in the Animal Experimentation Laboratory of the Biochemistry Department of the Federal University of Pernambuco (UFPE), under constant temperature (22 ± 2 °C), artificial lighting that provided a cycle of 12 h of light and 12 h of darkness, and access to food and water ad libitum. The experiments were approved by the ethics committee for animal use at UFPE (no. 109/2023).

### 2.4. Analgesic Activity of DEAQ

The anti-nociceptive activity of DEAQ was evaluated through formalin-induced nociception and acetic acid induced nociception. Mice were divided into six groups (n = 6 per group) for each assay, with separate batches of animals used for the acetic acid-induced writhing and formalin tests. The test groups received DEAQ at doses of 25, 50 and 100 mg kg^−1^ p.o.; the control group received 0.9% saline solution i.p., while the standards groups were treated with morphine 10 mg kg^−1^ i.p. or indomethacin 20 mg kg^−1^ i.p.

#### 2.4.1. Acetic Acid-Induced Writhing Test

The animals received the treatment 30 min prior to intraperitoneal administration of the phlogistic agent (0.8% acetic acid diluted in saline). For nociception analysis, the number of abdominal contortions in the period of 5–15 min after acetic acid injection was counted [18].

#### 2.4.2. Formalin Induced Nociception

After 60 min of treatment administration, 20 μL of formalin 2.5% (v/v) in saline solution was injected into the right paw of each animal. To measure the activity, the time (in seconds) of licking of the animals was recorded in two different time intervals after formalin injection. The first interval (0–5 min) represents the first phase, or neurogenic phase, and the second interval (15–30 min) refers to the inflammatory phase [18].

### 2.5. Mechanism of Action of the Antinociceptive Effect

The mechanistic studies of the antinociceptive effect of DEAQ were conducted using the formalin-induced nociception model to investigate the involvement of the opioid pathway in its antinociceptive activity. For that, independent animals were used and divided into experimental groups (n = 6 per group) for each assay, and DEAQ was administered orally, while all other treatments were administered via the intraperitoneal route (i.p.). The experimental sets shared the same control group (PBS, i.p.).

#### 2.5.1. Investigation of the Role of the Opioid Pathway in the Antinociceptive Effect

The animals were randomly assigned to six experimental groups (n = 6/group): a group treated with PBS (control, intraperitoneally—i.p.), a group treated with naloxone (2 mg kg^−1^, i.p.), a group treated with morphine (10 mg kg^−1^, i.p.), a group treated with DEAQ (100 mg kg^−1^, p.o.), a group pretreated with naloxone (2 mg kg^−1^, i.p.) 15 min before administration of morphine (10 mg kg^−1^, i.p.), and a group pretreated with naloxone (2 mg kg^−1^, i.p.) 15 min before administration of DEAQ (100 mg kg^−1^, p.o.). Naloxone, a non-selective opioid receptor antagonist, was used to investigate the role of the opioid pathway in the antinociceptive effect induced by DEAQ. The formalin test was performed 60 min after administration of PBS or DEAQ and 30 min after administration of morphine, according to protocols previously described in the literature.

#### 2.5.2. Investigation of the Types of Opioid Receptors (μ, δ, and κ) Involved in the Antinociceptive Effect

To evaluate the possible involvement of the μ, δ, and κ opioid receptor subtypes in the antinociceptive effect of DEAQ, the animals were divided into experimental groups (n = 6/group): a group treated with PBS (control, i.p.), a group treated with CTOP (selective μ-opioid receptor antagonist; 1 mg kg^−1^, i.p.), a group treated with naltrindole hydrochloride (selective δ-opioid receptor antagonist; 1 mg kg^−1^, i.p.), a group treated with nor-binaltorfimine dihydrochloride (selective κ-opioid receptor antagonist; 1 mg kg^−1^, i.p.), as well as groups pretreated with each selective antagonist 15 min prior to DEAQ administration (100 mg kg^−1^, p.o.). Following the treatments, the formalin test was performed 60 min after administration of PBS, antagonists, or DEAQ. Partial or complete reversal of the extract’s antinociceptive effect in the presence of the antagonists was considered indicative of the involvement of the respective opioid receptor subtype.

### 2.6. Statistical Analysis

Statistical analysis was performed by one-way analysis of variance (ANOVA), followed by the Bonferroni or Dunnet test of variation. The data obtained were analyzed using GraphPad Prism^®^ version 11.0 and expressed as mean values with standard deviation (±SD).

## 3. Results

### 3.1. Quantification of Cayaponoside D2 by HPLC Analysis

In previous studies, based on the interpretation of MS and MS^2^ spectra obtained from HPLC-ESI-MS/MS analyses and supported by literature data, cayaponoside D2 was iden-tified as the major compound present in DEAQ [16]. The HPLC-DAD method described above was employed for the quantification of cayaponoside D2 in the aqueous extract of *D. emetocathartica* stems.

**Figure 1:**
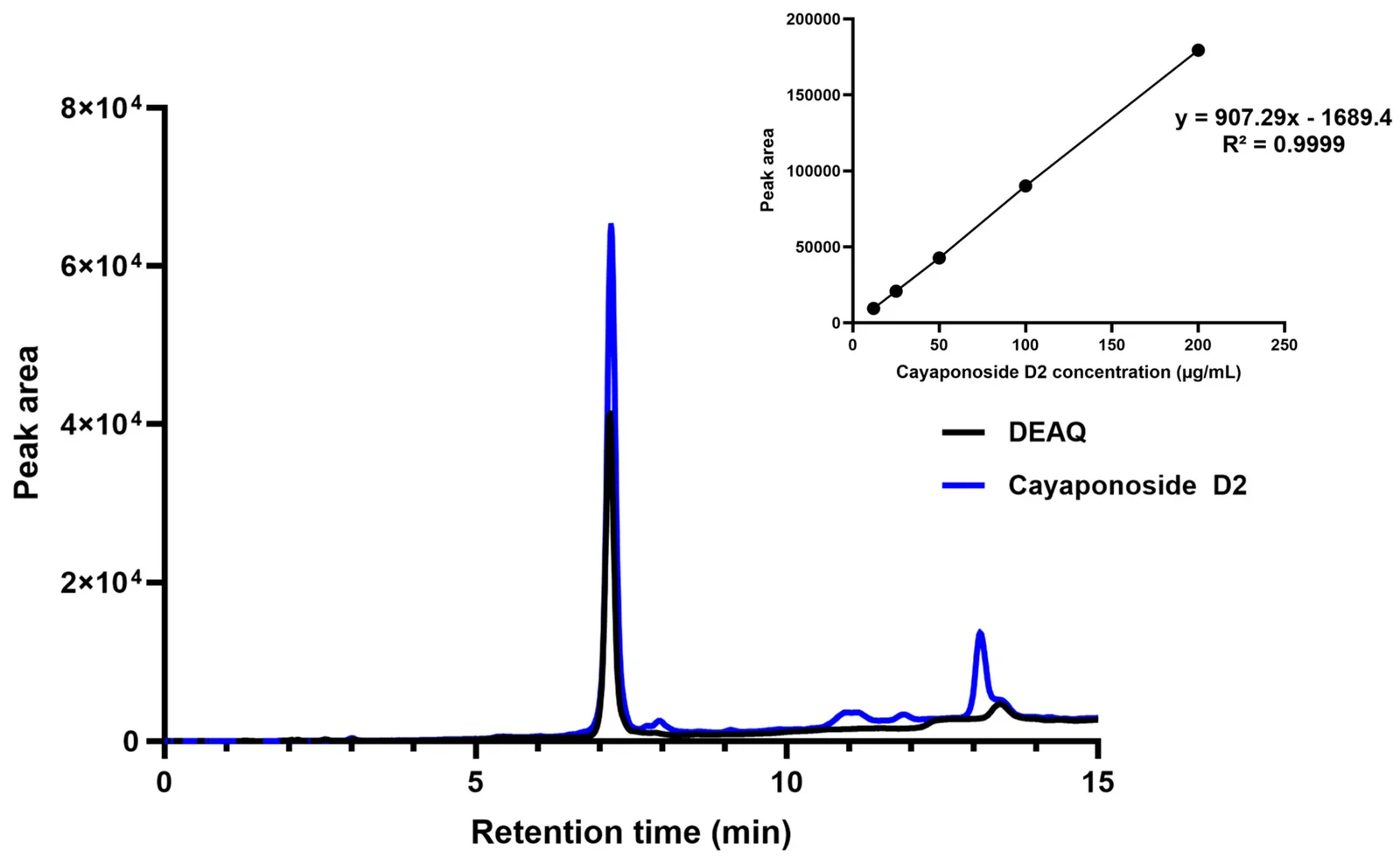
HPLC-DAD chromatograms and calibration curve for the quantification of cayaponoside D2 in DEAQ.

Figure 1 shows representative chromatograms of DEAQ and the cayaponoside D2 standard, with coincident peaks at 7.17 min corresponding to the target compound, as well as the calibration curve, which exhibited excellent linearity (R^2^ = 0.999), confirming the reliability of the method. Quantification was performed using the linear regression equation of the calibration curve (y = 907.29x − 1689.4). The content of cayaponoside D2 in the extract was determined to be 108 mg g^−1^ of dry extract.

### 3.2. Evaluation of Antinociceptive Activity

#### 3.2.1. Acetic Acid-Induced Whithing Test

**Figure 2:**
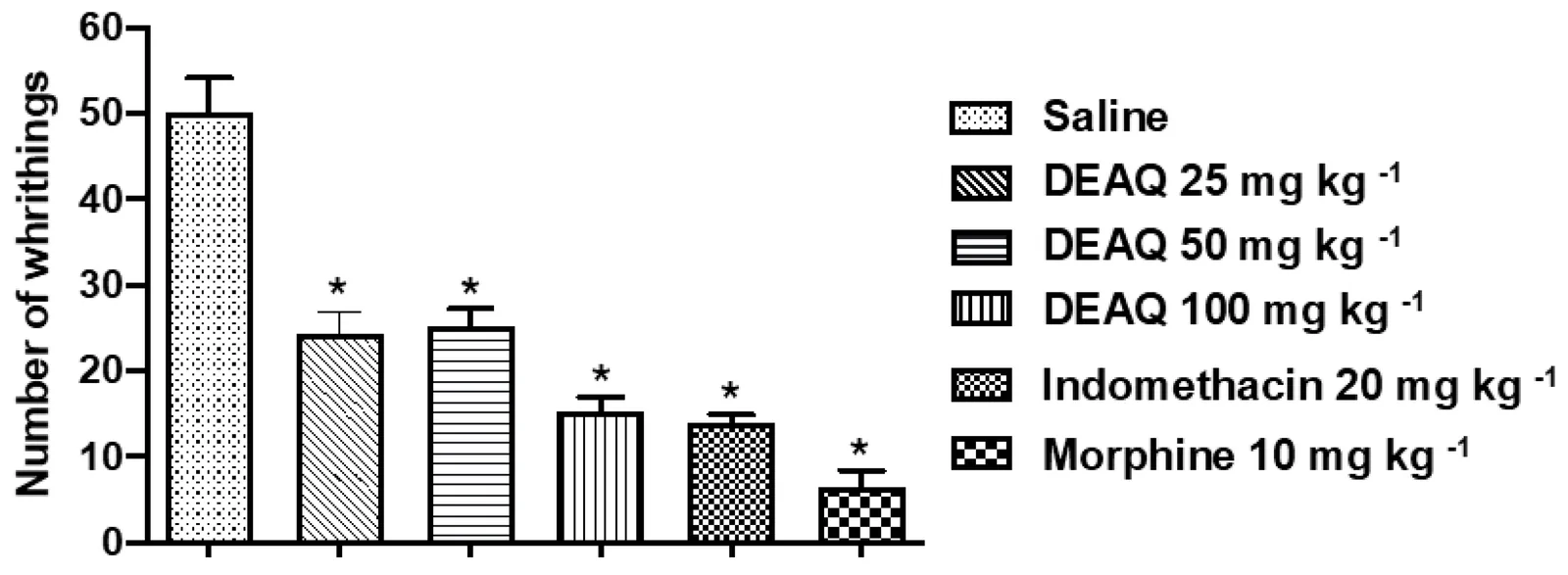
Antinociceptive effect of DEAQ on acetic acid-induced abdominal writhing. Values represent the mean ± SEM. * *p* < 0.05 compared to control, one-way analysis of variance (ANOVA) followed by Dunnett’s test.

The number of abdominal contortions was reduced with prior treatment using DEAQ. The groups treated with doses of 25, 50 and 100 mg kg^−1^ exhibited a reduction of 51.4, 49.6 and 69.6%, respectively. The group treated with morphine showed a reduction of 87%, while the group treated with indometacin showed a reduction of 72%, when compared to the group that received saline (Figure 2).

#### 3.2.2. Formalin-Induced Nociception

**Figure 3:**
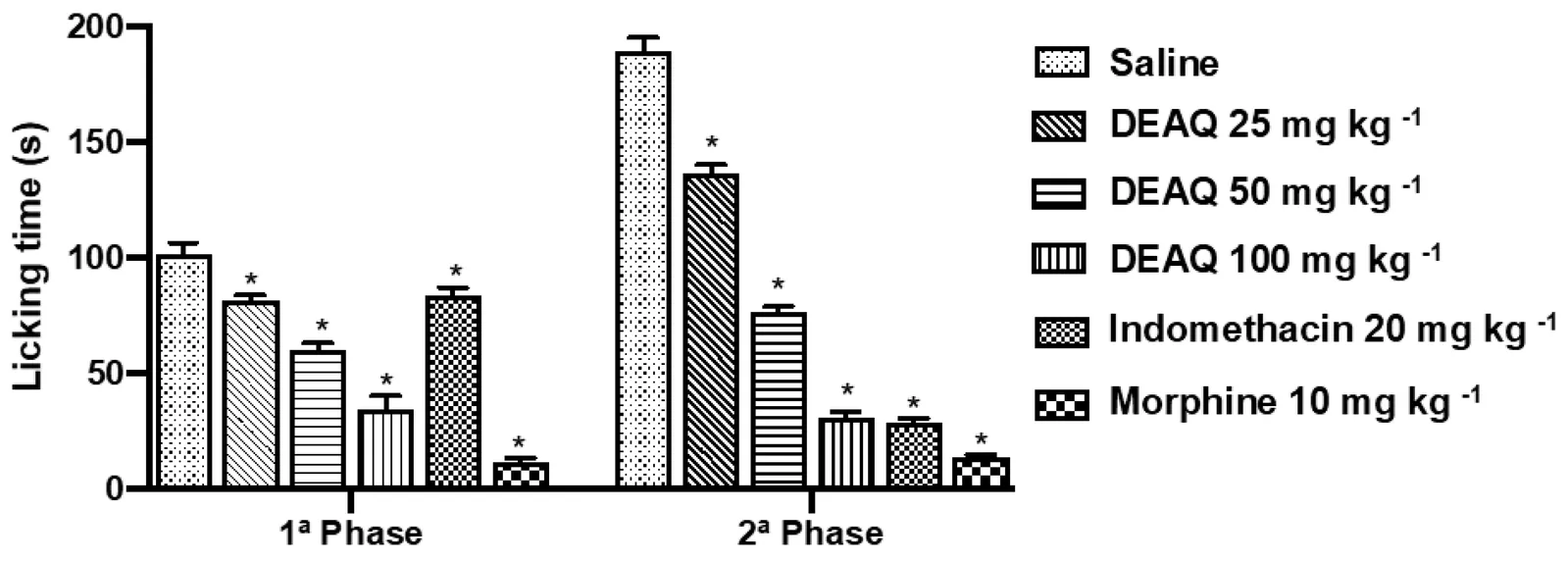
Antinociceptive effect obtained by administering DEAQ p.o. at doses of 25, 50 and 100 mg kg^−1^. * *p* < 0.05 compared to control, one-way analysis of variance (ANOVA) followed by Dunnett’s test.

During the first phase (neurogenic phase), the number of licks was reduced by 19.7%, 40.5% and 65.9% with DEAQ treatment at doses of 25, 50 and 100 mg kg^−1^, respectively, while treatment with morphine (10 mg kg^−1^) and indomethacin (20 mg kg^−1^) reduced licking by 88.4% and 17.5% after 5 min (Figure 3). In the second phase of the test (inflammatory phase), DEAQ doses of 25, 50 and 100 mg kg^−1^ reduced the paw licking time by 28.0, 59.7 and 83.6%, respectively. The indomethacin 20 mg kg^−1^ and morphine 10 mg kg^−1^ groups were able to reduce by 85% and 92.7% compared to the saline group (Figure 3).

### 3.3. Evaluation of Mechanism of Action of the Antinociceptive Effect

#### 3.3.1. Involvement of the Opioid Pathway in the Antinociceptive Effect

**Figure 4:**
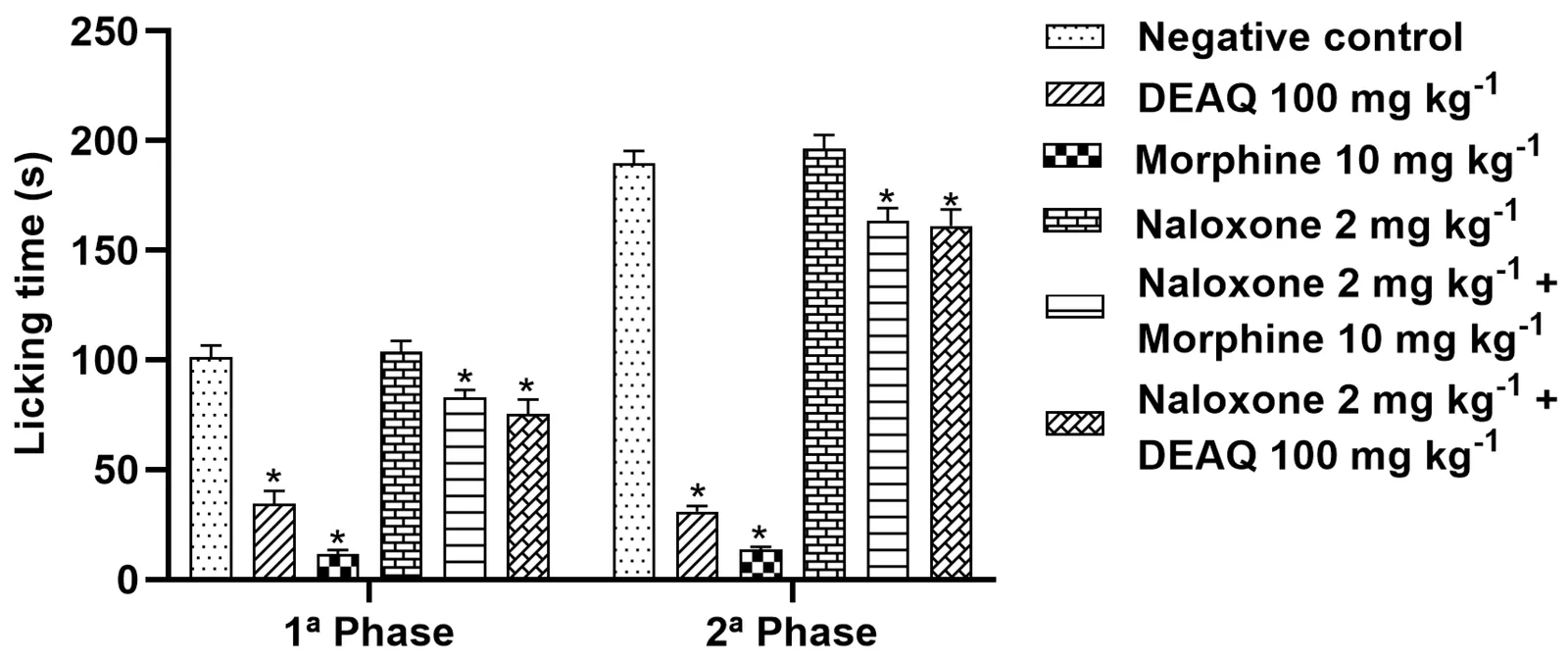
Investigation of the role of the opioid pathway in the antinociceptive effect. Data are presented as mean ± SEM (n = 6/group). * *p* < 0.05 compared to the negative control group; one-way analysis of variance (ANOVA) followed by Dunnett’s test. Naloxone partially reversed the antinociceptive effect of morphine and DEAQ, suggesting the involvement of the opioid pathway in the mechanism of action of the extract.

Figure 4 shows the licking times in the formalin test, evaluated during the 1st phase (neurogenic) and the 2nd phase (inflammatory), after different pharmacological treatments. An increase in licking duration reflects more pronounced nociceptive behavior, while a decrease indicates an antinociceptive effect. In the neurogenic phase, DEAQ 100 mg kg^−1^ reduced the licking time by 65.84% compared to the negative control group, corroborating the previously observed antinociceptive effects. The group treated with morphine 10 mg kg^−1^ showed a significant reduction in licking time, whereas the naloxone 2 mg kg^−1^ group did not exhibit a significant reduction compared to the control group. The groups treated with naloxone 2 mg kg^−1^ and 10 mg kg^−1^, as well as naloxone 2 mg kg^−1^ + DEAQ 100 mg kg^−1^, showed an increase in licking time of approximately sevenfold and twofold compared to the groups treated with morphine and DEAQ 100 mg kg^−1^, respectively. A similar pattern was observed in the inflammatory phase: pretreatment with naloxone 2 mg kg^−1^ significantly increased the nociceptive response in both the morphine-treated and DEAQ-treated groups.

#### 3.3.2. Involvement of Opioid Receptor Subtypes (μ, δ, and κ) in the Antinociceptive Effect

**Figure 5:**
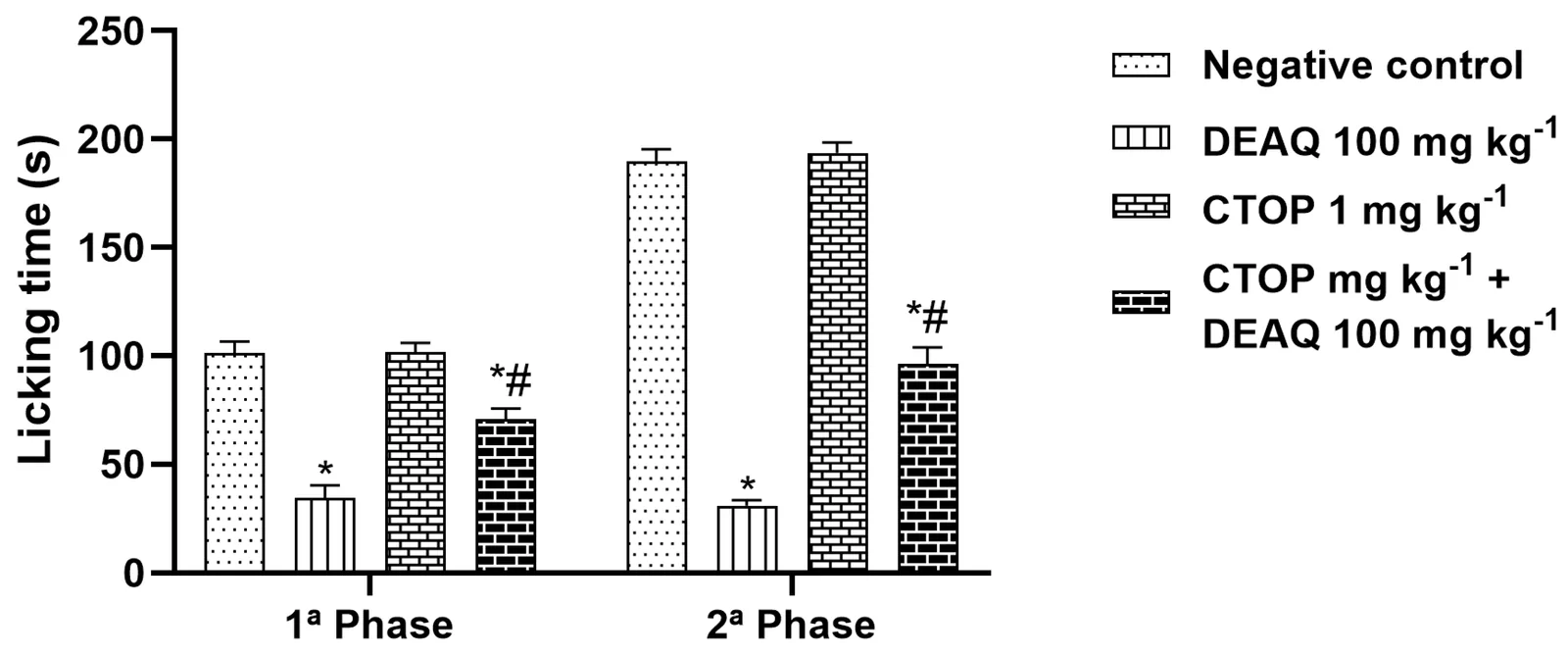
μ-Opioid receptor involvement in the antinociceptive effect. Data are presented as mean ± SEM (n = 6 per group). * *p* < 0.05 compared to the negative control group; # *p* < 0.05 compared to the DEAQ group, one-way analysis of variance (ANOVA) followed by Dunnett’s test. Pretreatment with CTOP partially reversed the antinociception induced by DEAQ, suggesting the involvement of μ-opioid receptors in the extract’s mechanism of action.

**Figure 6:**
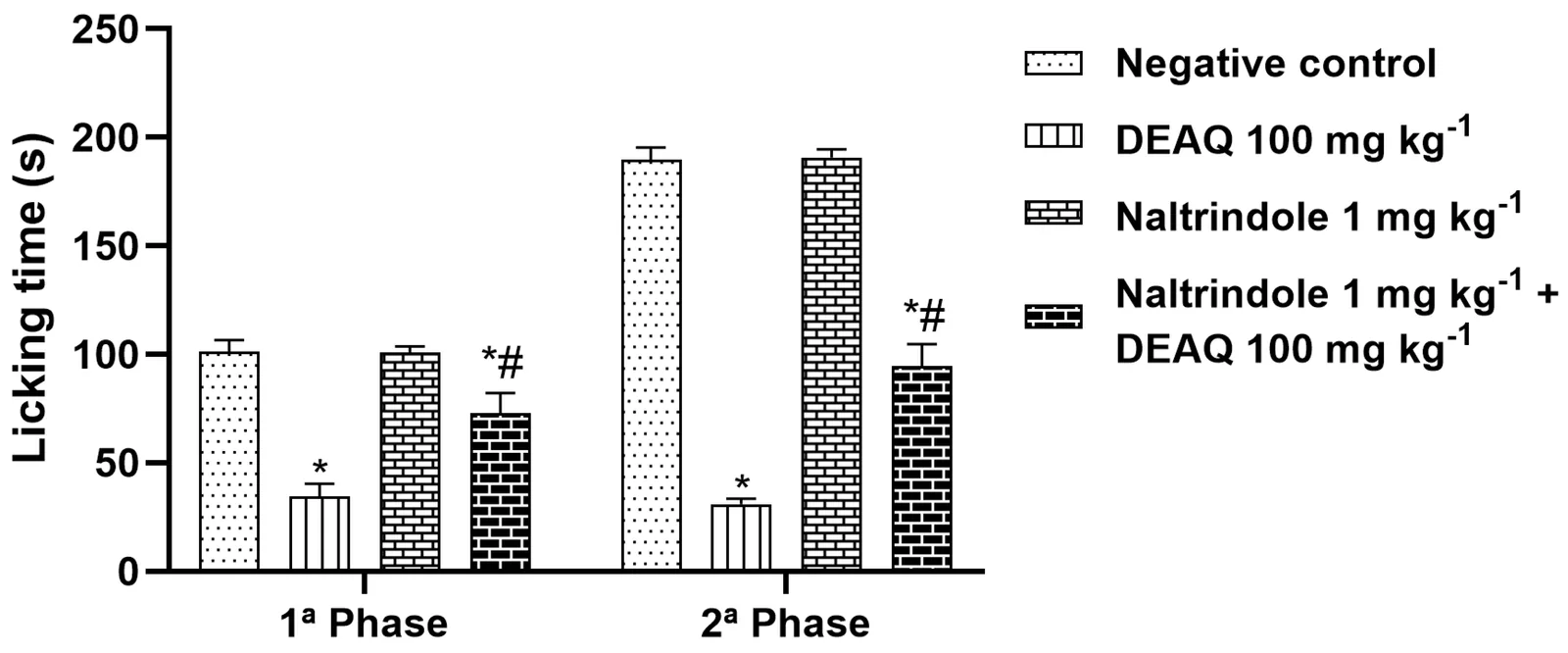
δ-Opioid receptor involvement in the antinociceptive effect. Data are presented as mean ± SEM (n = 6 per group). * *p* < 0.05 compared to the negative control group; # *p* < 0.05 compared to the DEAQ group, one-way analysis of variance (ANOVA) followed by Dunnett’s test. Pretreatment with naltrindole partially reversed the antinociception induced by DEAQ, suggesting the involvement of δ-opioid receptors in the extract’s mechanism of action.

**Figure 7:**
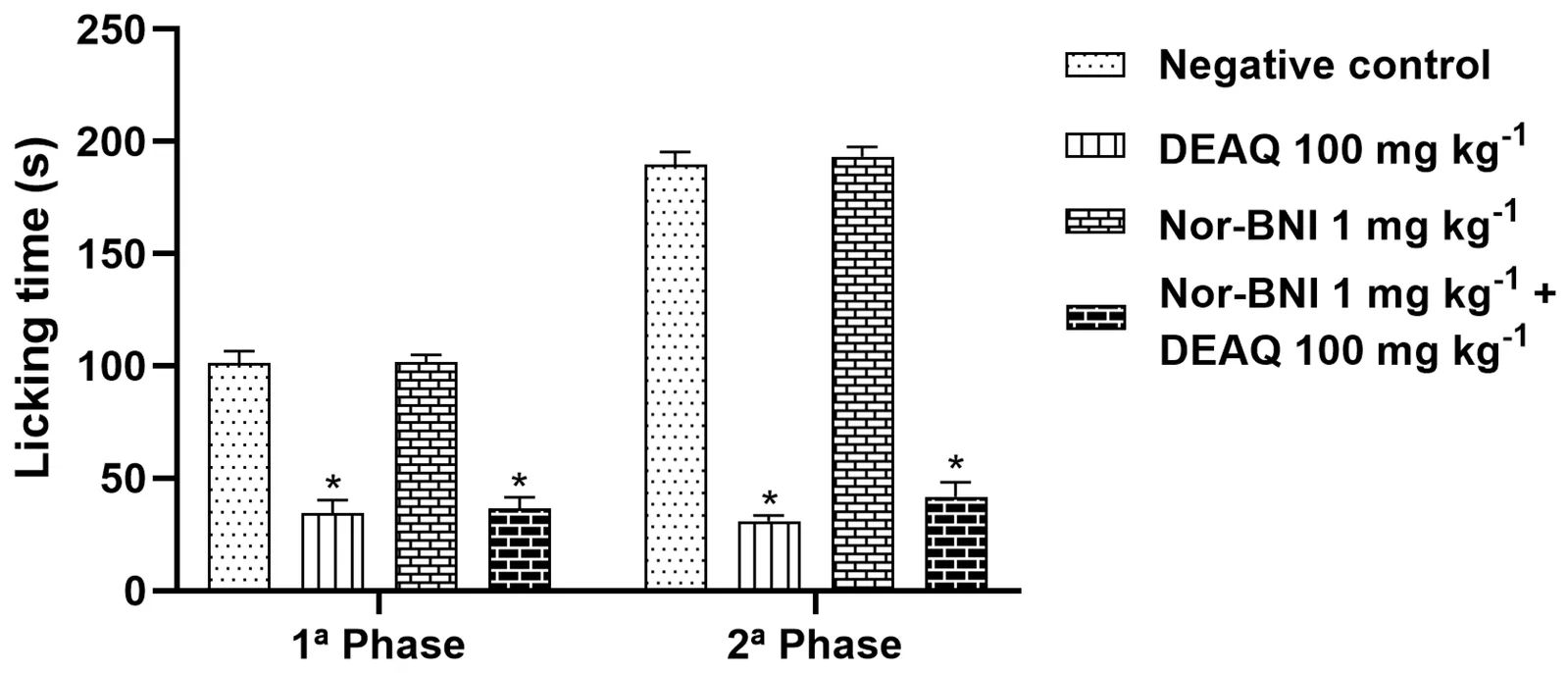
κ-Opioid receptor involvement in the antinociceptive effect. Data are presented as mean ± SEM (n = 6 per group). * *p* < 0.05 compared to the negative control group; Pretreatment with nor-BNI did not significantly reverse the antinociception induced by DEAQ, suggesting a lack of meaningful involvement of κ-opioid receptors in the extract’s mechanism of action.

Figure 5, Figure 6 and Figure 7 illustrate the effects of pretreatment with selective μ, δ, and κ opioid receptor antagonists on the antinociceptive activity of DEAQ in the formalin test. In Figure 5, treatment with CTOP 1 mg kg^−1^, a selective μ-opioid receptor antagonist, did not produce a significant change in licking time compared to the negative control group. However, pretreatment with CTOP 1 mg kg^−1^ prior to DEAQ administration 100 mg kg^−1^ resulted in an approximately twofold increase in licking time compared to the group treated with DEAQ 100 mg kg^−1^ alone. A similar pattern was observed in the inflammatory phase. CTOP 1 mg kg^−1^ alone did not significantly affect licking time, whereas the combination of CTOP 1 mg kg^−1^ and DEAQ 100 mg kg^−1^ led to a significant increase in the nociceptive response compared to the DEAQ-treated group. These findings suggest a partial involvement of μ-opioid receptors in the antinociceptive effect of DEAQ.

In Figure 6, treatment with naltrindole 1 mg kg^−1^, a selective δ-opioid receptor antagonist, did not result in a significant change in licking time compared to the negative control group. However, pretreatment with naltrindole 1 mg kg^−1^ prior to DEAQ administration 100 mg·kg^−1^ led to an approximately twofold increase in licking time relative to the group treated with DEAQ 100 mg kg^−1^ alone, following a pattern closely similar to that observed for μ-opioid receptors. A comparable effect was observed during the inflammatory phase. Naltrindole 1 mg kg^−1^ administered alone did not significantly affect licking time, whereas its combination with DEAQ 100 mg kg^−1^ produced a significant increase in the nociceptive response compared to the DEAQ-treated group. These results indicate a partial involvement of δ-opioid receptors in the antinociceptive effect of DEAQ.

In Figure 7, treatment with nor-BNI 1 mg kg^−1^, a selective κ-opioid receptor antagonist, did not result in a significant change in licking time compared to the negative control group. In contrast to the findings observed with μ- and δ-opioid receptor antagonists, pretreatment with nor-BNI 1 mg kg^−1^ prior to DEAQ 100 mg kg^−1^ administration did not result in a significant increase in licking time compared to the group treated with DEAQ 100 mg kg^−1^ alone. The values remained comparable to those observed in the DEAQ-treated group in both phases of the test. In the second phase, the group treated with nor-BNI alone showed licking times similar to the control group, whereas the nor-BNI + DEAQ group exhibited an antinociceptive response comparable to that observed with DEAQ administered alone, with no significant reversal of the effect.

## 4. Discussion

Previous studies on the chemical characterization of the aqueous extract of *D. emetocathartica* stems have demonstrated the presence of cayapanoside D2 as the major constituent, which may be related to anti-inflammatory properties evaluated in vivo models without inducing toxic effects under the experimental conditions evaluated [10]. This compound belongs to the class of glycosylated cucurbitacins, with a 29-norcucurbitane skeleton, and exhibits various reported pharmacological activities, including anti-inflammatory, antitumor, and antinociceptive effects. This class of compounds has been associated with pain control mainly through the modulation of inflammation [19,20].

In order to assess its analgesic capacity, DEAQ was used in abdominal writhing tests and formalin-induced nociception test. The injection of acetic acid into the peritoneal cavity causes irritation through the release of various inflammatory mediators, such as histamine, bradykinin, substance P and prostaglandins [21]. Another important factor in the perception of pain induced by acetic acid is the participation of TNF-α in the process of hypernociception [22]. Other cucurbitacin-producing plants from the same family have shown analgesic capacity in vivo, such as *Cucumis ficifolius* [23], *Cucurbita maxima*, *C. sativus* [24], and *Apodanthera glaziovii* [25]. Some cucurbitacins may act by selectively inhibiting some prostaglandins and decreasing the levels of TNF-α in the inflamed tissue and, consequently, attenuating nociception. Thus, the anti-nociceptive activity observed can be partially explained by the decreased levels of TNF-α found in the carrageenan-induced peritonitis test.

The formalin test is considered to be biphasic as it provokes nociception through the activation of two distinct pathways. The first phase, known as neurogenic, occurs through stimulation mainly of Aδ nerve fibers. The inflammatory phase, or late phase, is characterized by increased stimulation of C fibers by inflammatory mediators such as cytokines and prostaglandins [3]. The results indicate that DEAQ reduces pain perception in both phases, with a more pronounced effect during the inflammatory phase. This activity can be attributed, at least in part, to cayaponoside D2, which was quantified in DEAQ at a concentration of 108 mg per gram of dry extract (Figure 1). These findings reinforce the predominance of this compound in the aqueous extract of the stems from *D. emetocathartica*, supporting its relevance as a major contributor to the observed pharmacological effects. Similarly, results obtained by oral administration of the dichloromethane phase of *W. ebrectata* [26] and the aqueous extract from *C. ficifolius* [23] showed a greater predominance of antinociceptive activity during the second phase, which is attributed to the presence of cucurbitacins in both extracts tested. Based on the results obtained by the antinociceptive tests carried out, it is suggested DEAQ demonstrates analgesic activity by suppressing one or more factors present during the inflammatory process. An evaluation of the antinociceptive mechanism of action indicates that DEAQ exerts a pronounced effect on the opioid pathway, initially suggesting a profile similar to that of an agonist, comparable to that of morphine. This interpretation is supported by the results presented in Figure 4, which shows licking times in the formalin test for groups treated with DEAQ, morphine, and also in the presence of naloxone, a selective morphine inhibitor, demonstrating that DEAQ has an effect similar to that of morphine with opioid receptor-mediated analgesic action. However, pretreatment with naloxone prior to morphine administration partially increased nociceptive behavior compared to the morphine group, indicating partial reversal of the opioid analgesic effect. A similar result was observed in the naloxone + DEAQ group, in which licking times increased compared to the group treated with DEAQ alone during the neurogenic phase.

In the second phase of the test, primarily related to the inflammatory component and central sensitization, a similar pattern was observed. DEAQ significantly reduced licking time compared to the negative control, demonstrating a significant antinociceptive effect on inflammatory pain as well. Morphine again showed the greatest inhibition of nociception. Pre-treatment with naloxone caused a partial increase in the pain response in both the morphine group and the DEAQ group, indicating the involvement of the opioid pathway in the extract’s mechanism of action. Overall, the data suggest that DEAQ has a significant antinociceptive effect during the neurogenic and inflammatory phases of the line-form test. The partial reversal of this effect by naloxone indicates that the analgesic activity of the extract involves, at least in part, mechanisms mediated by opioid receptors. The involvement of specific opioid receptor subtypes was further evidenced by the use of selective antagonists. The effects of pretreatment with CTOP and naltrindole, selective antagonists of μ- and δ-opioid receptors, respectively, on the antinociceptive activity of DEAQ in the formalin test (Figure 5 and Figure 6) both promoted a significant increase in licking time compared to the group treated with DEAQ alone, indicating a partial reversal of the antinociception induced by the extract, since the values still remained lower than those of the negative control. In the second (inflammatory) phase, a similar pattern was observed in both treatments, indicating a significant increase in the pain response when compared to the DEAQ group, suggesting that the blockade of μ- and δ-opioid receptors partially attenuated the analgesic effect of the extract.

On the other hand, the effects of pretreatment with nor-BNI, a selective κ-opioid receptor antagonist, on the antinociceptive activity of DEAQ in the formalin test (Figure 7) did not result in a significant increase in licking time compared to the group treated with DEAQ alone in both phases of the test. These results demonstrate that there was no change in the antinociceptive response compared to DEAQ administered alone, with no significant reversal of the effect. Taken together, the results suggest that κ-opioid receptors do not play a significant role in the antinociceptive mechanism of action of DEAQ, since the selective blockade induced by nor-BNI did not significantly alter the effects of the extract.

The substances cayaponoside A1, cayaponoside B4, and cayaponoside D, isolated from the roots of *Siolmatra brasiliensis* (Cucurbitaceae), have demonstrated antinociceptive activity involving cholinergic and opioid pathways, suggesting that cucurbitane-type saponins may exert analgesic effects through opioid-mediated mechanisms [20]. Similarly, the results obtained in the present study indicate that DEAQ, an extract in which cayaponoside D2 is the major constituent, produces antinociceptive effects involving μ- and δ-opioid receptors.

Furthermore, the broad distribution of opioid receptors at peripheral and central levels may suggest that DEAQ exerts its effects through opioid pathways at both sites. Although knowledge regarding the ability of saponins to cross the blood–brain barrier remains limited, evidence suggests that the central effects of these compounds may be associated with their physicochemical properties, metabolic conversion into more lipophilic aglycones, the potential role of the glycosidic moiety as a prodrug, or the involvement of specific transport mechanisms [27,28,29]. Therefore, the participation of central opioid pathways in the antinociceptive activity of DEAQ cannot be excluded.

In addition to its potential role in the antinociceptive activity of DEAQ, the quantification of cayaponoside D2 in the extract represents an important step toward its chemical standardization, providing a potential marker for quality control and future technological development. Although the present study does not establish cayaponoside D2 as the sole compound responsible for the observed antinociceptive effect, its identification and quantification contribute to a better understanding of the relationship between the chemical composition and biological activity of DEAQ. These findings may support future investigations focused on extract optimization, reproducibility, safety, and efficacy, which are essential aspects for the development of herbal products with potential therapeutic applications.

Overall, these findings provide relevant evidence that DEAQ exhibits opioid-mediated antinociceptive activity and reveal important insights for the development of further studies aimed at exploring in detail the chemical and pharmacological potential of *D. emetocathartica*.

## 5. Conclusions

The evaluation of antinociceptive activity demonstrated that DEAQ effectively reduced pain perception in animals during both the neurogenic and inflammatory phases of the formalin test, with a more pronounced inhibitory effect observed in the inflammatory phase. Furthermore, the involvement of the opioid system in its mechanism of action is strongly supported by pharmacological antagonism assays, which indicate that the antinociceptive effect is predominantly mediated by μ-opioid receptors, with an additional contribution from δ receptors and minimal involvement of κ receptors. The partial reversal of the effect observed following pretreatment with opioid antagonists further indicates that the mechanism of action of DEAQ is not exclusively opioid-dependent, supporting the hypothesis of a multimodal pharmacological profile. These findings reinforce the pharmacological potential of the aqueous extract from *D. emetocathartica* in antinociceptive therapy and provide scientific evidence supporting its ethnopharmacological use in pain management. In this context, the extract emerges as a promising candidate for the development of plant-derived therapeutic agents for pain treatment, contributing to the advancement of safer and more effective analgesic strategies.

## Data Availability

The original contributions presented in this study are included in the article Further inquiries can be directed to the corresponding author.

## Abbreviations

The following abbreviations are used in this manuscript:

DEAQ	*Doyerea emetocathartica* aqueous extract
ANOVA	Analysis of Variance
TNF-α	Tumoral Nuclear Factor—alpha
SEM	Standard Error of The Mean
